# Sensory and Volatile Flavor Analysis of Beverages

**DOI:** 10.3390/foods10010177

**Published:** 2021-01-17

**Authors:** Alice Vilela

**Affiliations:** CQ-VR Chemistry Research Centre, Department of Biology and Environment, School of Life Sciences and Environment, University of Trás-os-Montes and Alto Douro (UTAD), 5001-801 Vila Real, Portugal; avimoura@utad.pt; Tel.: +351-259-350-973; Fax: +351-259-350-480

Humans have used their senses to evaluate food for several thousands of years. Since so many phytotoxins and bacterial metabolites are bitter and sour, humanity probably used sensory evaluation since before *Homo sapiens* were human. Historically, the evaluation of beverage flavor, both sensorially and chemically, focused on the presence or absence of defects (e.g., clarity, color, volatile acidity, etc.). This frequently involved the use of “expert” tasters who evaluated the appearance, color, odor, taste, and mouthfeel to arrive at an overall impression of the beverage “quality”, based on the absence of defects and the overall “balance” of sensory properties. Development of analytical methods in the 19th century linked the measurement of sensory defects to beverage composition, and served as a basis for many early laws aimed at protecting beverage “quality”.

Beverage quality in the beverage industry is heavily influenced by ingredient flavor quality. Complexity is a term widely used in beverage degustation and is considered a positive characteristic, and desirable in wines, beers, ciders, liquors, and even in non-alcoholic drinks such as juices, coffee, and teas/plant infusions. But what is complexity? Some authors state that complexity is an associative perception of multiple elements, especially from the synergy of several individual compounds, and it is possible to consider the number of flavor compounds that can be detected as a complexity indicative.

To evaluate a beverage’s complexity, it is possible to choose chemical analysis, for the identification of flavor compounds. It is also possible to evaluate the human perception of the synergy through sensory analyses. The relation between both analyses, chemical and sensory, in beverages, is an extensive research area in the beverage industry.

This special issue is composed of thirteen different types of work—one review paper, and twelve research articles—written by a group of international researchers to provide up-to-date research on the different dimensions of the vast research world of sensory and volatile flavor analysis of beverages.

Rosend et al. [1] from Estonia, studied the possible application of freshly pressed apple juice and juice concentrate, as a substrate for cider fermentation. Estonian apples are a great source for tasty ciders. The results showed that in the samples fermented with the concentrate, the yeasts consumed less fructose. The formation of long-chain fatty acid esters increased with the use of reconstituted juice concentrate, while the differences in off-flavor formation could not be determined. Overall, the use of the concentrate could be considered efficient for cider fermentation, although some nutritional supplementation might be required to support the vitality of yeast.

Consumers’ acceptability and perceived sensory attributes are topics of interest all over the sensory research world. Heo et al. [2] from Korea and the USA, developed an interesting work about cold brew coffee, using data from 120 consumers, which evaluated 13 cold brew coffee samples and checked sensory attributes using the check-all-that-apply (CATA) method. With this study, they were able to specify the characteristics of this beverage and consumers’ segmentation, using acceptability.

Coffee was also the subject of study for Korean researchers, Lee et al. [3]. The authors investigated the nutritional, physicochemical, and sensory characteristics of coffee brewed with conventional and high-oleic peanut extracts. Compared to normal coffee, peanut coffee displayed a large diversity in amino acid composition. Using e-tongue analysis, peanut coffee showed an increased intensity of sweetness and umami taste, and a decrease in bitterness. This study provides informative data in extending the application of peanut to coffee and developing innovative coffee, which besides being aromatically pleasant is, also, nutritious.

The frequency of the attribute citation method, a well-known sensory technique, was applied by Nanou et al. [4], a group of researchers from Greece and France. The authors aimed at investigating the sensory aroma profiles of white wines of the indigenous Greek grape varieties Assyrtiko, Malagousia, Moschofilero, and Roditis. They also used robust sensory data analysis techniques, such as correspondence analysis (CA) and cluster analysis, to investigate the sensory space of wines. Despite common characteristics found within the Greek grape-varieties, some samples of different varieties exhibited overlapping profiles, and in some cases, samples of the same variety were quite different from each other.

The new descriptive method based on free description Pivot© profile [5] was used by Longo et al. [6] aiming to identify potential color components, and volatile and sensory attributes that could discriminate Pinot noir wines from five Australian winegrowing regions. The sensory analysis showed that wines from the Mornington Peninsula presented different sensory descriptors than those from Adelaide Hills, indicating that regionality is a strong driver of aroma typicity of Australian wines.

The impact of two different winemaking practices on the chemical and sensory complexity of Pinot Blanc wines from South Tyrol (Italy), from grape pressing to the bottled wine stored for nine months, was studied by Dupas de Matos et al. [7]. In this interesting article, new chemical markers of Pinot blanc were identified—astilbin and trans-caftaric acid differentiated the wines according to the vinification; S-glutathionylcaftaric acid correlated with the temporal trends.

Two international research groups, with the participation of Professor Malfeito-Ferreira from Portugal and his co-workers from Brazil, proposed two fascinating articles, where consumers and emotional responses were the main themes. Souza-Coutinho et al. [8] developed a wine tasting sheet, including sensory and emotional responses, to simplify the recognition of fine white wines by consumers. A group of 104 consumers evaluated five white wines with different sensory characteristics using an improved emotional wine tasting sheet. The emotions and sensations most frequently associated with white wines were obtained through the Check-All-That-Apply (CATA) test. At the end of the work, the identification of fine wine attributes and their incongruity with emotional responses could be used as a valuable instrument by professionals, to explain the different wine styles to consumers. Romano et al. [9] studied the up-to-date subject of consumer’s acceptance of organic wines, which are usually linked to the unpleasant perception of off-flavors. Blind tasting sessions of wines of both types of production (organic and conventional), with and without off-flavors, using a tasting panel that included experienced individuals of several nationalities were performed. These researchers found that off-flavors and their unpleasantness functioned as a cue to identify wines supposed to be organic by experienced tasters. However, consumers depreciated wines with unpleasant flavors, regardless of their mode of production.

Port wine, the ultimate expression of the Portuguese Demarcated Douro Region’s (DDR’s) history, cultural heritage experience, and art was also the theme for one of the works presented. Vilela et al. [10] developed a tawny port wine-like fragrance, the first according to the literature. This fragrance could be used in sensory marketing in restaurants or even in personal-use products. Interestingly, from the seven aromatic chemical compounds used to initially construct the fragrance, only three (benzaldehyde, sotolon, and vanillin) remained until the end of the experiment and were enough to produce the tawny port wine-like fragrance, much appreciated by young and middle-aged adults.

A ternary cross-modal interaction aroma–sweetness–viscosity was evaluated by Bertelsen et al. [11] from Denmark, in two beverage matrices, water and apple nectar. An interesting article that addressed the new industry challenge of sugar reduction in food and beverage products. The perceptual effects of vanilla aroma (0–1 mL/kg), sucrose (2.5–7.5% w/w), and pectin (0–0.3% w/w) were studied in both matrices. The results indicated that pectin, a natural heteropolysaccharide that occurs in the cell walls of fruits and vegetables, could be used to improve the mouthfeel in sugar-reduced beverages, without compromising the taste or aroma perception.

Additionally, aiming to evaluate the sensory characteristics and volatile compounds that affect consumers’ acceptance of rice liquors Heo et al. [12] from Korea, performed a noteworthy work where a total of 80 consumers of different ages evaluated 12 yakju samples and 11 trained panelists performed a descriptive analysis. Solvent-assisted flavor evaporation-gas chromatography-mass spectrometry analysis revealed 120 volatile compounds in the yakju samples. The multi-factor analysis (MFA) correlation map showed that ethyl propanoate, ethyl-2-hydroxy-2-methylbutanoate, methyl 2-furoate, γ-butyrolactone, 4-ethoxycarbonyl-γ-butyrolactone, hawthorn odor, apple flavor, grape flavor, and sweet taste were positively correlated with young consumers’ overall acceptance. Additionally, a negative correlation with overall acceptance was found in 1,3-butanediol, 2,3-butanediol, and 1,1-diethoxy-3-methyl butane.

Malongane et al. [13] from South Africa, investigated the descriptive sensory analysis and volatile compounds of the bush, special, Honeybush, and rooibos tea, and their blends. Once South Africa had a traditional heritage of using indigenous herbal teas. The necessity for herbal teas driven by the functional health benefits associated has far beaten global supply. Therefore, this is an important study, not only in terms of academic knowledge but also for industrial purposes. The results demonstrated that rooibos and Honeybush tea had an overall sweet-caramel, honey-sweet, perfume floral, and woody aroma while bush tea and special tea depicted green-cut grass, dry green herbal, and astringent/dry mouthfeel. Moreover, compounds identified in this study can be valuable markers for discriminative herbal tea sensory characteristics.

Finally, a review article was written by Vilela et al. [14]. The authors made a review aiming to present the current state of the art of beverage fragrance biotechnology, including recent advances in sensory and sensor methodologies, such as e-noses and e-tongues, and advanced statistical techniques for sensory data analysis.

In summary, the Special Issue “Sensory and Volatile Flavor Analysis of Beverages” demonstrates that the sensory science applied in beverages evolved and can be applied in different beverage matrices, using different tasting panels, from experts to simple consumers. Moreover, when conjugated with chemometric methods, it is a useful tool for analyzing beverage quality.

I am greatly indebted to the authors who generously shared their scientific knowledge and experience with others through their contribution to this special issue.
